# Large eddy simulation of cough jet dynamics, droplet transport, and inhalability over a ten minute exposure

**DOI:** 10.1063/5.0072148

**Published:** 2021-12-15

**Authors:** Hadrien Calmet, Kiao Inthavong, Ambrus Both, Anurag Surapaneni, Daniel Mira, Beatriz Egukitza, Guillaume Houzeaux

**Affiliations:** 1 Department of Computer Applications in Science and Engineering, Barcelona Supercomputing Center (BSC-CNS), Barcelona, Spain; 2 Mechanical & Automotive Engineering, School of Engineering, RMIT University, Melbourne, Australia

## Abstract

High fidelity simulations of expiratory events such as coughing provide the opportunity to predict the fate of the droplets from the turbulent jet cloud produced from a cough. It is well established that droplets carrying infectious pathogens with diameters of 1–5 μm remain suspended in the air for several hours and transported by the air currents over considerable distances (e.g., in meters). This study used a highly resolved mesh to capture the multiphase turbulent buoyant cloud with suspended droplets produced by a cough. The cough droplets' dispersion was subjected to thermal gradients and evaporation and allowed to disperse between two humans standing 2 m apart. A nasal cavity anatomy was included inside the second human to determine the inhaled droplets. Three diameter ranges characterized the droplet cloud, <5 μm, which made up 93% of all droplets by number; 5 to 100 *μ*m comprised 3%, and >100 μm comprising 4%. The results demonstrated the temporal evolution of the cough event, where a jet is first formed, followed by a thermally driven puff cloud with the latter primarily composed of droplets under 5 *μ*m diameter, moving with a vortex string structure. After the initial cough, the data were interpolated onto a more coarse mesh to allow the simulation to cover ten minutes, equivalent to 150 breathing cycles. We observe that the critical diameter size susceptible to inhalation was 0.5 μm, although most inhaled droplets after 10 min by the second human were approximately 0.8 μm. These observations offer insight into the risk of airborne transmission and numerical metrics for modeling and risk assessment.

## I. INTRODUCTION

Coughs are a dramatic respiratory activity implicated as a primary source for airborne transmission of viral laden droplets. Aerosols generated from a cough become airborne leading to increased susceptibility to transmission to others nearby. Recent evidence has highlighted the potential airborne spread of cough droplets containing viruses,^1,2^ where the risk of its transmission is likely when there are inadequate ventilation and prolonged exposure to respiratory particles produced from coughing, sneezing, singing, or shouting. Aerosol generation from coughs has inspired many studies to investigate the droplet-laden cough jet and puff dynamics through experimental and computational methods,^3–6^ as well as review articles of the fluid dynamics of respiratory droplets^7–9^ and mitigation strategies for reducing viral transmission.^10,11^

While many studies applied lower-fidelity models (e.g., RANS turbulence models), the Large Eddy Simulation (LES) and Direct Numerical Simulation (DNS) approaches provide greater details. Studies applying LES include^12^ which used a model containing 38 × 10^6^ elements modeled for 10 s of physical time and showed that at a higher room temperature, the cough jet and droplet penetration is shorter;^13^ investigated the cough jet development with and without the influence of co-flow air;^14^ applied high-fidelity LES on a computational grid that was three orders of magnitude finer than most studies; and^15^ modeled cough jet plume development in six ventilation cases and found that a vast majority of droplets remained suspended within the puff after the liquid evaporated. Studies using the DNS approach include^16^ which detailed the initial jet stage and a dissipative phase over which turbulence intensity decayed; and^17^ showed the effects of indoor ventilation and ambient conditions on the virus-laden droplet lifetime. These studies focused on the cough jet in detail, and the longest simulation time covered 10 s.

In general, findings from the literature showed that droplet diameters wielded significant influence,^3,4,18,19^ with small droplet diameters (e.g., less than 5 *μ*m) being the most significant for airborne transmission.^20^ Bourrouiba *et al.*^21^ combined experimental and theoretical analysis of the fluid dynamics of a cough and observed multiphase turbulent buoyant clouds with suspended droplets of various sizes. The cough jet acts as a carrier phase in transporting the droplets. Wei and Li^22^ characterized a human cough structure as a two-stage jet and suggested that a vortex at the head of the jet played an important role in enhancing droplet transport. Cough droplets were dispersed primarily by the initial jet cough,^21,22^ followed by a vortex ring structure^23^ which moves faster than the associated thermal puff cloud.^24,25^

The ambient environmental conditions also influenced droplets where the droplets were airborne for longer in warmer and more humid settings.^26,27^ Further studies showed that the cough exhibited pulsatility^28,29^ and that the droplet dispersion and penetration of the entire cloud were hindered by increased pulsatility. However, the penetration of droplets emanating from a secondary or tertiary expulsion was enhanced due to acceleration downstream by the vortex structure.^30^ By incorporating the multi-parameters that influence the airborne transmission, several studies provided a platform for evaluating exposure risk.^8,29,31,32^ While these new findings provide an improved understanding of airborne transmission of the virus, Mittal *et al.*^8^ highlighted the challenges faced in mitigating its spread by identifying three critical components for airborne contagion transmission. These were the host, the environment, and the susceptible person, which combine to evaluate the minimum number of droplets required by inhalation to become infected by the virus.

In this work, we expand existing studies with two primary aims. The first focused on resolving the cough jet and characterizing the vortex ring structure that transports the droplets downstream. The second was the investigation of the droplet dispersion over ten minutes to provide a medium-term exposure risk between two humans standing 2 m apart. A highly resolved mesh was used to establish the cough jet before the fluid, and particle data were interpolated onto a reduced mesh, and the simulation was performed with larger time steps to determine the temporal variation in droplet dispersion between the two humans. This made it possible to simulate the dispersion over 10 min.

## II. GEOMETRY AND MESH GENERATION

**FIG. 1.**
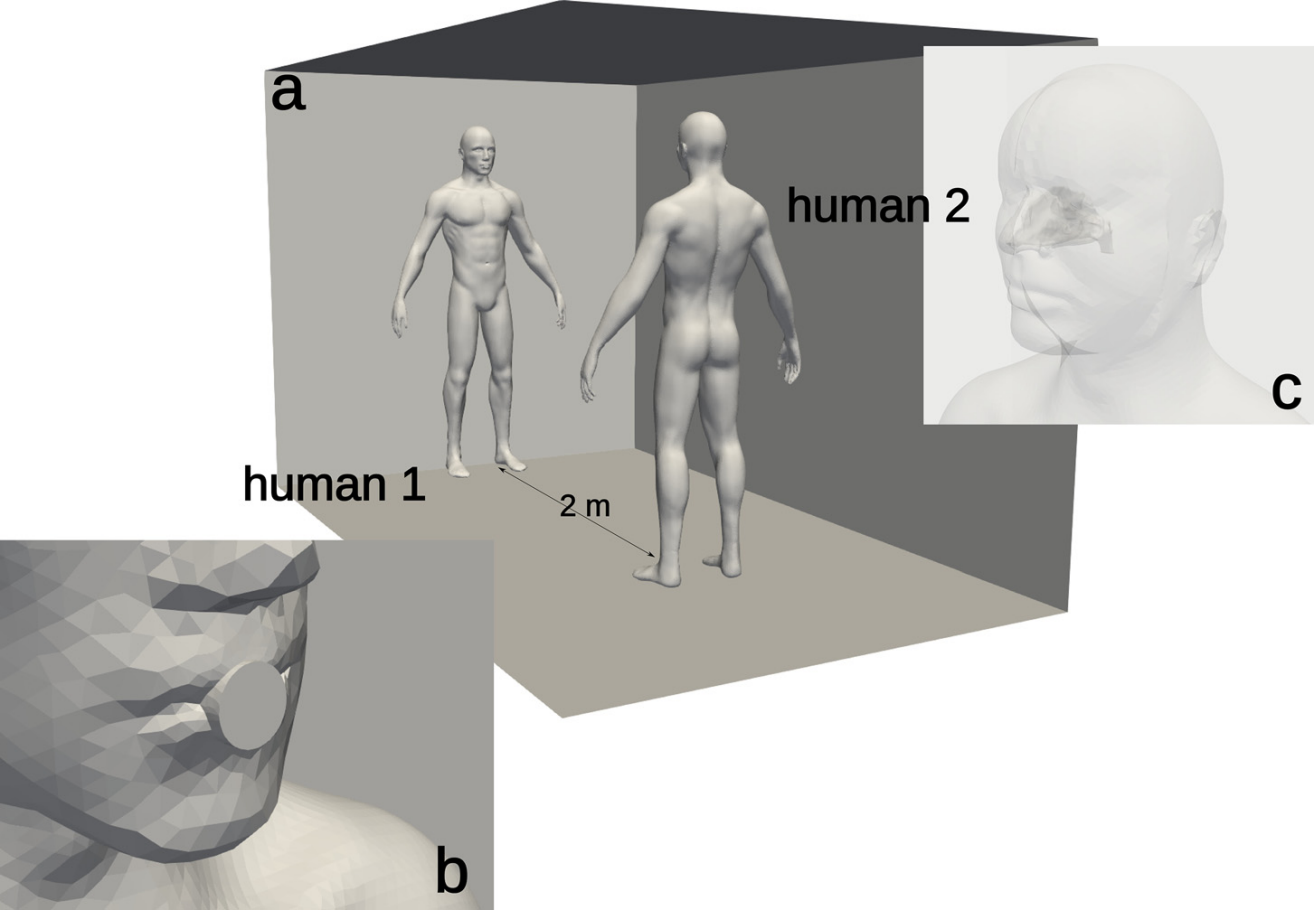
Geometry of the room (a) and details of the mouth opening area for human 1 (b) and geometry of the upper airways of human 2 (c).

The geometry model consisted of two humans standing face-to-face in a room with a floor size of 2 × 3 m^2^ and a height of 3 m, see Fig. 1(a). The height of both humans was 1.7 m. The first human (labelled “human-1”) produced a cough from the mouth, and the second human (labelled “human-2”) was modeled with the nasal cavity performing respiratory breathing at 4 s per inhalation–exhalation cycle. The nasal cavity was reconstructed from computed tomography scans of a 48-year-old male subject, retrospectively collected from a large hospital database (details are available in Refs. 33 and 34).

**FIG. 2.**
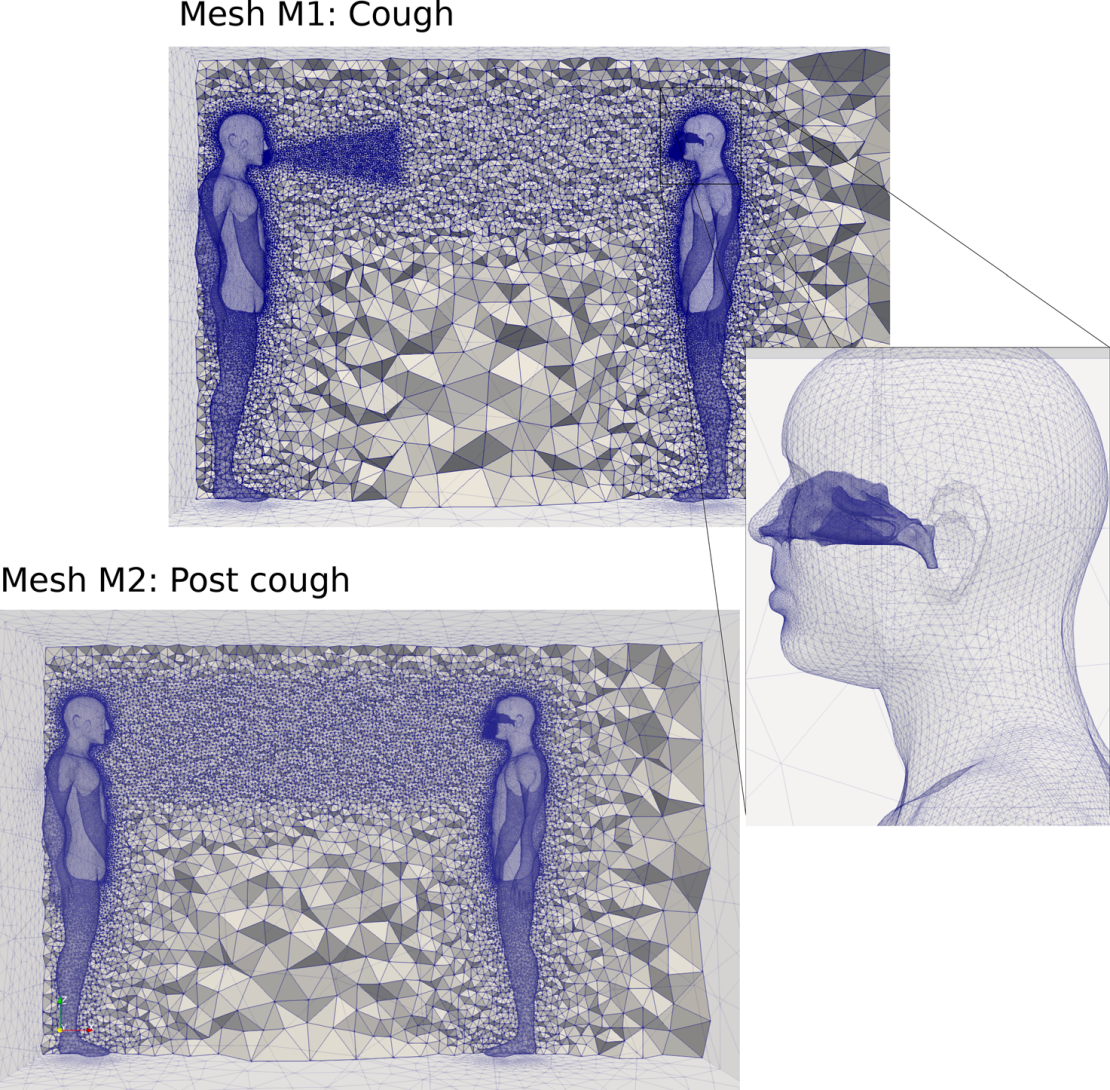
Grid generation topology for M1 and M2 with details of the nasal cavity for human 2.

Two different meshes were generated for this study. The first mesh (labelled “M1”) was created to resolve the cough jet plume behavior. The second mesh (labelled “M2”) was optimized to resolve the droplet/aerosol transport for 10 min in the room. The computational method applies low-dissipation finite element algorithms with an explicit time-stepping scheme; thus, the coarsening of the jet region in mesh M2 allows for larger time steps, making the simulation of 10 min feasible. To accurately capture the features of the jet produced by the cough from human-1, a mesh refinement zone located in the front of the mouth was created (see Fig. 2). A fine mesh resolution was also applied to the nasal cavity geometry for human-2 and in the vicinity of the breathing region. A mesh refinement around the external bodies was performed to reproduce the thermal plume from the bodies.

Mesh M1 applied a conical refinement region around the cough jet region. Following the description of Pope,^35^ the cone angle was 12° and the length of the cone to capture the developing jet region was 0.6 m $\left(30D_{m}\right)$ where *D_m_* = 20 mm is the mouth diameter. The self-similarity region is characterized by the jet distance of ( $30D_{m}$ to $100D_{m}$). For computational efficiency, only the developing jet region had a mesh refinement with an element size of 5 mm (see Table I) which produced 3.7 × 10^6^ elements. In the M2 mesh model, the refined jet cone region was removed, and a mesh refinement was performed between the two bodies, (element size decreased from 4 to 2 cm). The mesh generation was performed with ANSYS ICEM CFD v170 software.

**TABLE I.** Summary of different mesh resolutions and simulation parameters with *N_N_*: number of nodes, *N_E_*: number of elements, ΔT: time step, Δ_*J*_: grid size in the jet core, Δ_*B*_: grid size between the two bodies, and time: the duration of the simulation.

Mesh	$N_{N}(\times 10^{6})$	$N_{E}(\times 10^{6})$	ΔT (μs)	$\Delta_{J}\;(\text{mm})$	$\Delta_{B}\;(\text{mm})$	Time (s)
M1 (cough)	0.7	3.7	5	5	40	1
M2 (post-cough)	0.6	3.6	300	0	20	600

## III. BOUNDARY CONDITIONS

An initial flow field was established by modeling 4 s on mesh M1 that covered one breathing cycle from human-2, while allowing the body temperatures to generate a thermal plume. The body surface temperatures were set to 34 °C, while the room temperature was 23 °C with a relative humidity of 20%. Evaporation from the surface of the bodies was neglected by applying a zero gradient boundary condition for the vapor mass fraction on the body surface. The resulting velocity, vapor mass fraction, and temperature fields were used to initialize the cough simulation. The walls and the floor are also treated with Dirichlet boundary conditions, equal to the initial conditions of the room, while a zero gradient boundary condition was applied at the outlet.

**FIG. 3.**
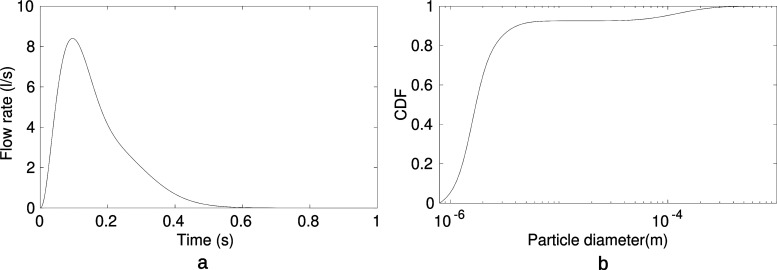
(a) Volume flow rate in liters per second (l/s) generated during the cough as function of time.^36^ (b) Cumulative distribution function of the droplet diameter during the cough event.^37^

The cough flow profile from human-1 is given in Fig. 3(a), which was based on Gupta *et al.*^36^ Three parameters characterized the cough dynamics: cough peak flow rate, peak velocity time, and cough expiration volume. Droplets emitted during the cough were characterized based on Johnson *et al.*,^37^ producing a tri-modal droplet size distribution [Fig. 3(b)]. The three modes are associated with three distinct locations of droplet formation: one occurring deep in the lower respiratory tract (bronchioles), another in the throat (larynx), and a third in the upper respiratory tract (oral cavity), resulting in the BLO tri-modal model. The parameters of the cough droplet size distribution are detailed in the Appendix section.

The volume flow rate profile in liters/second (l/s) generated by the cough^36^ was imposed as a Dirichlet condition at the mouth of human-1 which was simplified to a circular shape diameter, *D *=* *2 cm with an area of 3.14 cm^2^ following the range of results of the mouth opening area during a cough Gupta *et al.*^36^ The Reynolds number at the peak flow rate based on the circular diameter was *Re_d_* = 44 000. A zero-traction outflow condition (where the surface is free from external stress) was imposed at the upper room ceiling to represent an open environment, while the remaining walls were treated as a no-slip boundary condition.

**FIG. 4.**
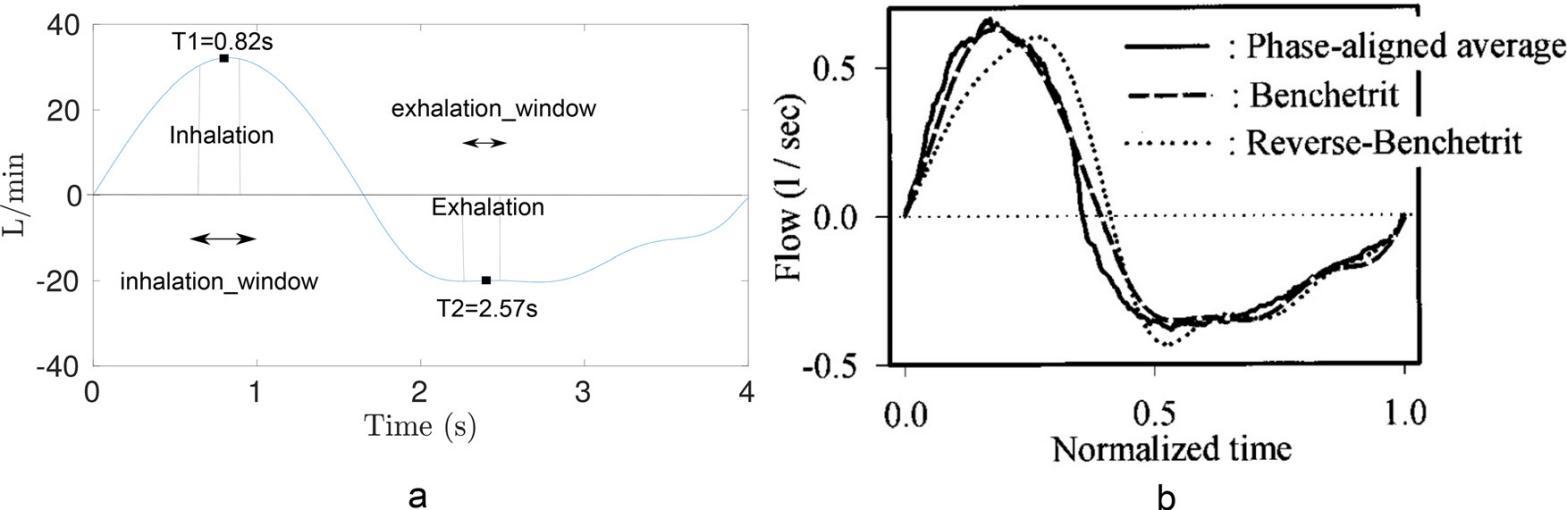
(a) Generated transient respiratory cycle and average periods used in this study. (b) Processed results image taken from Sato and Robbins.^39^ A tidal volume of 550 ml was selected based on statistical analysis of the data in Benchetrit *et al.*^38^ which involved 16 subjects measured with an average of 50 breaths.

For the inlet conditions at the mouth, Dirichlet boundary conditions were imposed for temperature and vapor mass fraction. The temperature was set to 34 °C, while the water vapor mass fraction was imposed such that a relative humidity value of 80% was produced. The breathing profile for human-2 was created from Benchetrit *et al.*,^38^ Sato and Robbins^39^ shown in Fig. 4, which measured breathing profiles of sixteen healthy adult subjects over two studies separated by four to five years to test whether their resting pattern of breathing was reproducible over time. In this study, a tidal volume of 550 ml was used over a breathing cycle of 4 s, made up of 1.65 s of inhalation, and 2.35 s of exhalation. The flow rate was converted to l/min and was defined by a sum of sine equations used in Ref. 40 and plotted in Fig. 4(a), given as

Inhalation: t=0.523 sin (1.9t) for *t *=* *0 to 1.65 s;

Exhalation: t=−0.3674 sin (1.429(t−1.65))−0.0351 sin (6.223(t−1.65))−0.0807 sin (3.572(t−1.65)) for *t *=* *1.65 to 4.00 s.

To avoid flow discontinuity where the flow changes directions at $t_{c}=1.65$ and *t_c_* = 4, mixing functions based on the hyperbolic tangent were used.

The droplets were introduced into the domain at the circular mouth opening at time t = 0.03 s and stopped at t = 0.22 s continuously at every Δt=0.5 ms. Droplet breakup, which is preceded by droplet deformation that could influence the drag, was not considered. However, this is limited to the initial cough region (near the mouth), where larger droplets were introduced with high velocities. The ambient humidity evaporates the droplets causing a decrease in the droplet diameter where the drag is altered and is a function of the particle Reynolds number. At the initial cough region near the mouth, we take an average velocity of 10 m/s for a droplet diameter of 100 *μ*m giving a moderate Weber number of 139, while downstream there are much smaller droplets due to evaporation. For a droplet diameter of 10 *μ*m moving at 1 m/s, the Weber number is low at 1.39, which suggests that droplet breakup is limited to the near mouth cough region only.

### A. Fluid solver

The governing equations of flow used an Eulerian–Lagrangian approach. The Eulerian description represented the fluid carrier phase, while the liquid droplets were modeled with the Lagrangian Particle Tracking (LPT) method. The equations modify the Eulerian solver to consider the individual droplet dynamics in the Lagrangian reference. The approach is applied in the LES framework using Favre-filtered equations to avoid modeling terms, including density fluctuations. Favre-filtering of a quantity ϕ is denoted by $\tilde{\phi }$, while Reynolds-average filtering is given by $\bar{\phi }$.

The LES filtered governing equations in the Eulerian solver include the continuity, momentum, enthalpy, and mass fraction of water in the gas phase for the low-Mach number limit.^41^ The latter is used to track the mixing fields between evaporated droplets and air. The system of equations is given by

$$\frac{\partial \bar{\rho }}{\partial t}+\nabla \cdot \left(\bar{\rho }\tilde{u}\right)={\bar{S}}_{C},$$ (1)

$$\frac{\partial \bar{\rho }\tilde{u}}{\partial t}+\nabla \cdot \left(\bar{\rho }\tilde{u}\tilde{u}\right)=-\nabla \cdot \bar{\tau_{M}}-\nabla \bar{p}+\nabla \cdot \left(\bar{\mu }\nabla \tilde{u}\right)+{\bar{S}}_{M},$$ (2)

$$\frac{\partial \bar{\rho }\tilde{h}}{\partial t}+\nabla \cdot \left(\bar{\rho }\tilde{u}\tilde{h}\right)=-\nabla \cdot {\bar{\tau }}_{h}+\nabla \cdot (\bar{\rho }\bar{D}\nabla \tilde{h})+{\bar{S}}_{H},$$ (3)

$$\frac{\partial \bar{\rho }\tilde{Y_{v}}}{\partial t}+\nabla \cdot (\bar{\rho }\tilde{u}\tilde{Y_{v}})=-\nabla \cdot {\bar{\tau }}_{Y_{v}}+\nabla \cdot \left(\bar{\rho }\bar{D}\nabla \tilde{Y_{v}}\right)+{\bar{S}}_{Y_{v}}.$$ (4)

For the set of equations (1)–(4), standard notation is used for all the quantities with $\bar{\rho },\;\tilde{u},\;\tilde{h},\;\tilde{Y_{v}},\;\tilde{D},\;\bar{p}$, and $\bar{\mu }$ representing the density, velocity vector, total enthalpy (sensible and chemical), mass fraction of evaporated droplets, diffusivity, pressure, and dynamic viscosity using filtered quantities. The *τ* term denotes the unresolved or subgrid terms related to the filtering operation and applies to the unresolved momentum flux ${\bar{\tau }}_{M}$, enthalpy flux ${\bar{\tau }}_{h}$ and water vapor mass fraction flux ${\bar{\tau }}_{Y_{v}}$. The subgrid viscous stress tensor is determined based on Stoke's assumption, and the turbulence contribution is obtained using the Boussinesq approximation.^42^ Heating due to viscous forces is neglected in the enthalpy equation, and the unresolved heat flux is modeled using a gradient diffusion approach.^43^ The modeling framework is closed by an appropriate expression for the subgrid-scale viscosity. The eddy-viscosity is obtained from the Vreman^44^ model using a constant $c_{k}=0.1$. The wall-adapting local-eddy viscosity model (WALE)^45^ was used for the subgrid-scale model. This model provided good results in previous simulations of respiratory airways being competitive compared with more computational demanding models like the dynamic Smagorinsky, see Ref. 46. The source terms appearing in the previous equations (*S_C_*, *S_M_*, *S_H_*, and $S_{Y_{v}}$) come from the coupling of the disperse phase with the gas phase. The closure for these terms is obtained by the integration of Eqs. (6), (11), and (12).

The set of equations is time integrated using an energy conserving Runge-Kutta explicit method for momentum and scalar transport, proposed by Capuano *et al.*,^47^ combined with an eigenvalue-based time step estimator.^48^ For more details about the LES method, see Ref. 49.

### B. Droplet model and cough droplet size distribution

The evaporating droplet cloud produced by the cough is modeled in the Lagrangian reference frame. Each droplet is characterized by its location, velocity, mass, and temperature. A system of ordinary differential equations is formulated and solved numerically to track the change in these properties. The kinematic model was responsible for monitoring the particle location and velocity and loosely coupled to the evaporation model that describes the heat and mass transfer between the gas phase and the droplets.

The droplets ejected in a coughing event originate from various mucosal tissues. Although they mainly consist of water, they also contain different nonvolatile species in multiple proportions, such as salts, proteins, and surfactants. After most of the initial water content evaporates, these nonvolatile components remain in the particle, forming the so-called droplet nuclei.

Treatment of a multi-component system is simplified by assuming the properties of pure water and imposing a lower limit to the evaporated droplet mass of 6% of the initial mass.^50^ This limit corresponds to approximately 40% of the initial diameter and is an adequate estimation for a wide range of environmental conditions.^50^

**FIG. 5.**
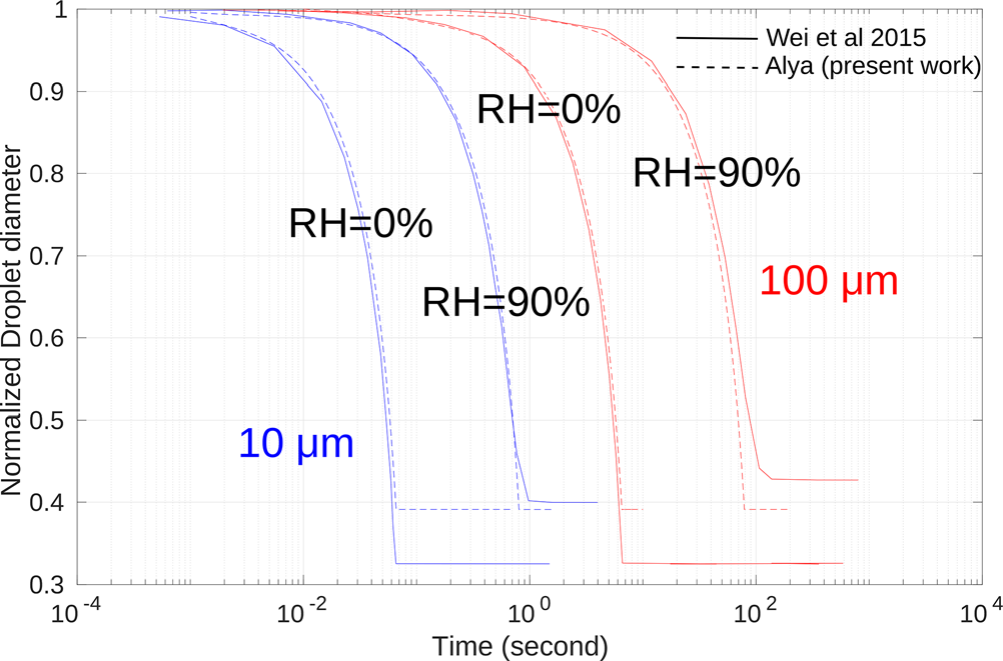
Evaporation of droplets under different relative humidity (RH), $d_{p}=10\;\mu m$, blue line; $d_{p}=100\;\mu m$, red line.

To validate the numerical framework of the droplet model, we simulated a single droplet evaporating under different ambient relative humidity conditions, studied by Wei *et al.*^51^ Figure 5 shows good agreement between their model and the present work with a limitation on the minimum droplet nucleus size. While the relative nucleus size in a multi-component approach is a function of the relative humidity of the environment and the initial droplet size, it is simply a constant in the present work. Nevertheless, this approach is sufficient to capture aerosol transport.

The initial droplet diameter is imposed such that the BLO tri-modal size distribution of Jonhson *et al.*^37^ is reproduced [Fig. 3(b)]. The size distribution is a superposition of three lognormal distributions corresponding to the three sites where respiratory droplets originate: the Bronchioles, the Larynx, and the Oral cavity. The B-mode is concentrated on O(1 μm) droplets, the L-mode corresponds to droplets of $1\;\mu m<d_{p}<20\;\mu m$, while the-O mode represents much larger droplets formed in the oral cavity in the range of $20\;\mu m<d_{p}<1000\;\mu m$.

The cumulative density function (CDF) was pre-evaluated on a logarithmically spaced grid in the validity range of the BLO model: $0.8\;\mu m\leq d_{p}\leq 1000\;\mu m$. The diameter of the next injected droplet was determined through a pseudo-random number *ξ* in the [0,1) interval and interpolating the diameter based on the inverse of the CDF:

$$d_{p}^{\mathrm{next}}=CDF^{-1}(\xi )\;\mathrm{with}\;\xi \in [0,1).$$ (5)

### C. Particle transport

The Lagrangian solver was used to predict individual droplet motion from coughing. The droplets were treated with a kinematic model, while the thermodynamic conditions of the droplet are given from heat and mass transfer models. The dominant forces considered in the kinematic modeling are the drag force and gravity, given by

$$\frac{du_{p}}{dt}=g-\frac{1}{2}\frac{\rho C_{d}A_{p,c}}{m_{p}}(u_{p}-u)|u_{p}-u|,$$ (6)

where $u_{p}$ is the particle velocity, **g** the gravitational acceleration, *C_d_* its drag coefficient (function of its Reynolds number), $A_{p,c}$ the particle cross-sectional area, and *m_p_* the particle mass. The gas velocity, **u**, and gas density, *ρ*, are obtained from the Eulerian equations. The particle transport equation takes into account buoyancy effects, which are included by adding a gravitational force.

The droplet advances its position $x_{p}$ in time from the known velocity determined by

$$\frac{dx_{p}}{dt}=u_{p}.$$ (7)

Assuming spherical droplets, the drag coefficient was computed as

$$C_{d}=\{\begin{array}{cc} 24/{\text{Re}}_{D}, & {\text{Re}}_{D}\leq 1,\\ 24(0.85+0.15Re_{D}^{0.687})/{\text{Re}}_{D}, & 1>{\text{Re}}_{D}\leq 1000,\\ 0.44, & \text{for}\;1000<Re_{D}, \end{array}$$ (8)

with

$$Re_{D}=\frac{\rho |u_{p}-u|2r_{p}}{\mu (T)},$$ (9)

where μ(T) is the dynamic viscosity of air at temperature *T* and *r_p_* is the particle radius.

Momentum exchange from the droplets to the air (gas phase) is determined by summing the forces from each droplet in a grid element and dividing by the element volume, *V*, [see Eq. (2)], which is obtained by

$$S_{M}=\frac{1}{V}\sum \left[\frac{\rho }{2}C_{d}A_{p,c}(u_{p}-u)|u_{p}-u|\right].$$ (10)

The mass and energy transfer can be described by the following set of equations:

$$\frac{dm_{p}}{dt}=k_{m}A_{p,s}\rho \;\text{ln}\;(1+B_{m}),$$ (11)

$$\frac{dT_{p}}{dt}=\frac{1}{m_{p}c_{p}}\left[A_{p,s}k_{h}(T-T_{p})+\frac{dm_{p}}{dt}h_{v}\right],$$ (12)

where *B_m_* is the Spalding mass number given by

$$B_{m}=\frac{Y_{f,i}-Y_{f,s}}{1-Y_{f,i}},$$ (13)

where *m_p_* is the mass of the droplet, $A_{p,s}$ is the surface area of the liquid droplet, *k_m_* is the mass transfer coefficient, *ρ* the gas density, *c_p_* is the liquid specific heat, *k_h_* is the heat transfer coefficient between the droplet and the gas, and *h_v_* is the latent heat of vaporization of the liquid.

By summing the particles in a cell we get a source term for the continuity and evaporated liquid phase ${\tilde{Y}}_{v}$ equation, see Eqs. (1) and (4), which is

$$S_{C}=S_{Z}=-\frac{1}{V}\sum \frac{dm_{p}}{dt},$$ (14)

where *V* is the cell volume. The source term for the energy equation [see Eq. (3)] is given as

$$S_{H}=\frac{1}{V}\sum \left[A_{p,s}k_{h}(T_{p}-T)-\frac{dm_{p}}{dt}(h_{v}+h_{l})\right],$$ (15)

where *h_l_* is the liquid specific enthalpy, and *h_v_* is the latent heat of vaporization of the liquid.

Mass and heat transfer coefficients between liquid and gas are described with analogous empirical correlations. The mass transfer coefficient, *k_m_*, is described by

$$k_{m}=\frac{\text{Sh}D_{lg}}{L};\;\text{Sh}=2+0.6{\text{Re}}_{D}^{\frac{1}{2}}{\text{Sc}}^{\frac{1}{3}},$$ (16)

where Sh is the Sherwood number, *D_lg_* is the binary diffusion coefficient between the water vapor and the surrounding gas (air), *L* is a length scale (equal to the droplet diameter), Re_*D*_ is the Reynolds number of the droplet (based on the droplet diameter, *D*, and the relative air-droplet velocity), and Sc is the Schmidt number $\nu /D_{lg}$ ( ν=μ/ρ is the kinematic viscosity).

An analogous relationship exists for the heat transfer coefficient,

$$k_{h}=\frac{\text{Nu}k}{L};\;\text{Nu}=2+0.6{\text{Re}}_{D}^{\frac{1}{2}}{\text{Pr}}^{\frac{1}{3}},$$ (17)

where Nu is the Nusselt number, *k* is the thermal conductivity of the gas, and Pr is the Prandtl number.

The exchange of mass and energy between liquid droplets and the surrounding gases is computed droplet by droplet. After the temperature of each droplet is computed, the appropriate amount of vaporized liquid is added to the given mesh cell, and the cell gas temperature is balanced based on the energy exchange between the droplet and the surrounding.

## IV. RESULTS

### A. Cough

The coughing event can be described as a multiphase turbulent buoyant cloud with suspended droplets of various sizes. The current definition of the droplet cloud is viewed mostly in a binary manner of either a cloud of “droplets” or “aerosols” where the WHO (World Health Organisation) defines diameter of >5 μm as droplets, and <5 μm as aerosols. Recent work by Randall *et al.*^52^ reviewed the historical foundation of these concepts, and we add another category to this dichotomy which is a “mix of aerosols and droplets” ( $5\;\mu m<d_{p}<100\;\mu m)$.

**FIG. 6.**
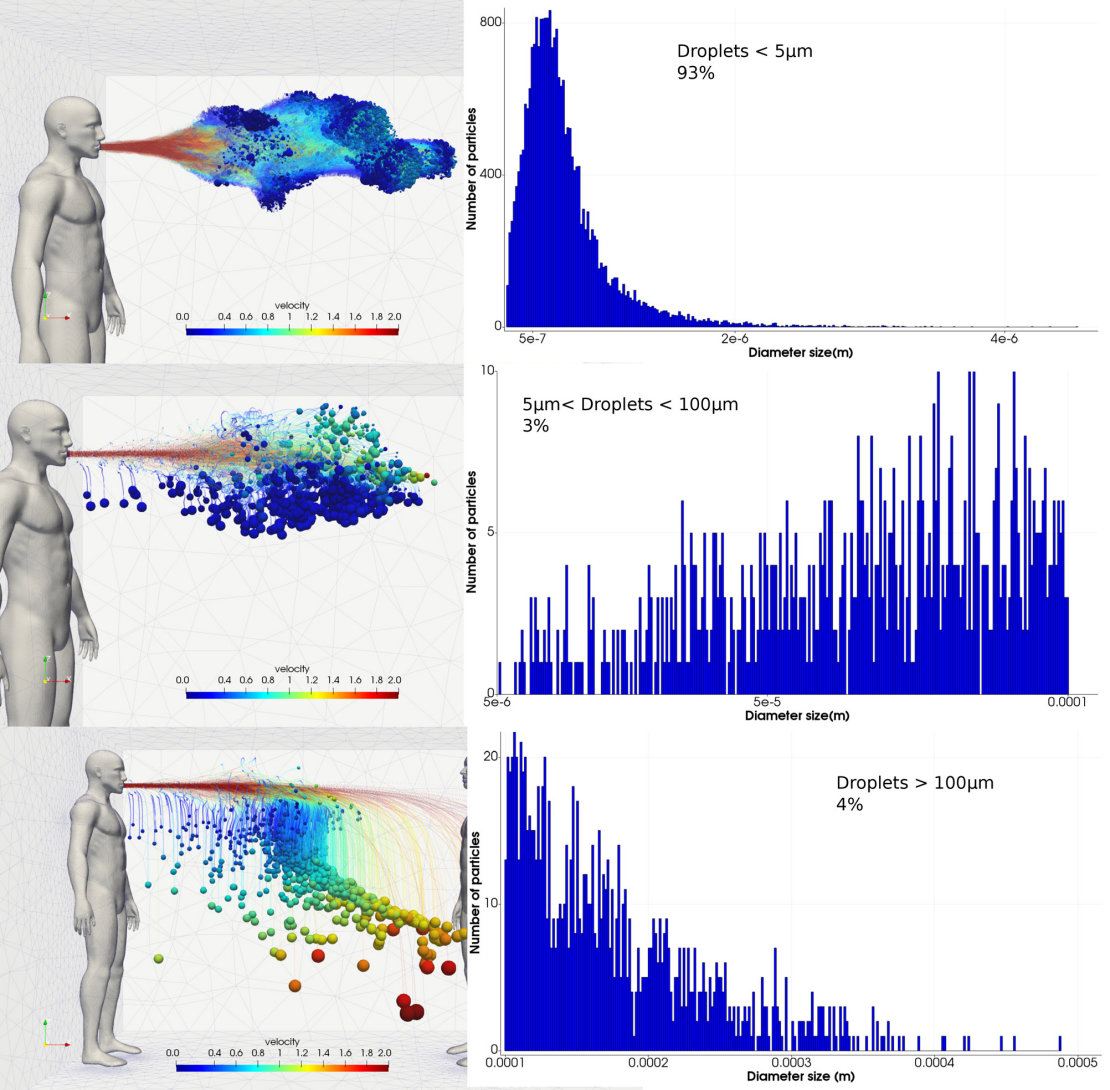
Description of the classification with on the left side the trajectory of the droplets colored according to their velocity and on the right side the corresponding size distribution and total fraction.

Based on this new trichotomy, we separate the emitted cough droplets into three categories to better describe the behavior of the droplet sizes as a continuum embedded in a turbulent exhalation cloud trapping and transporting them. Figure 6 shows this classification at 1 s after the cough, where the droplet trajectories are shown on the left and its corresponding size distribution on the right.

The cough after 1 s primarily consisted of droplets of <5 μm, which made up 93% of all droplets by number, which formed the cloud puff, and move horizontally unaffected by gravity. Droplets in the mixed range of 5–100 *μ*m comprised 3%, while in the larger diameter range of >100 μm there was 4%. The size distribution for the small droplets ( <5 μm) exhibited a well-defined pattern where the mean value was approximately 0.5 μm. However, the size distribution for the two other size categories exhibited a random pattern with no clear pattern, even when wider size bins were used. This compares with the tri-modal size distribution model that was found in Johnson *et al.*^37^ where the size distribution is a superposition of three lognormal distributions corresponding to the three sites where respiratory droplets originate: the bronchioles, larynx, and the oral cavity.

#### 1. Influence of thermal puff and droplets

The temporal evolution of the cough event can be described in two parts, where a jet is first formed, followed by a puff, which was described in Bourouiba *et al.*^21^ At 0.5 s, we observe that 90% of the droplets are embedded in the thermal puff cloud which are primarily composed of droplets under 5 *μ*m diameter. At these droplet diameters, the evaporation phenomenon has completed (within 0.5 s). We do not describe in detail the turbulent mechanisms during a cough as this is well explained in the recent literature.^16,30,53,54^ However, we note that the larger turbulent eddies that influence the particle transport are resolved through the LES approach which is transient. The particles are transported with the eddies with each small time step.

**FIG. 7.**
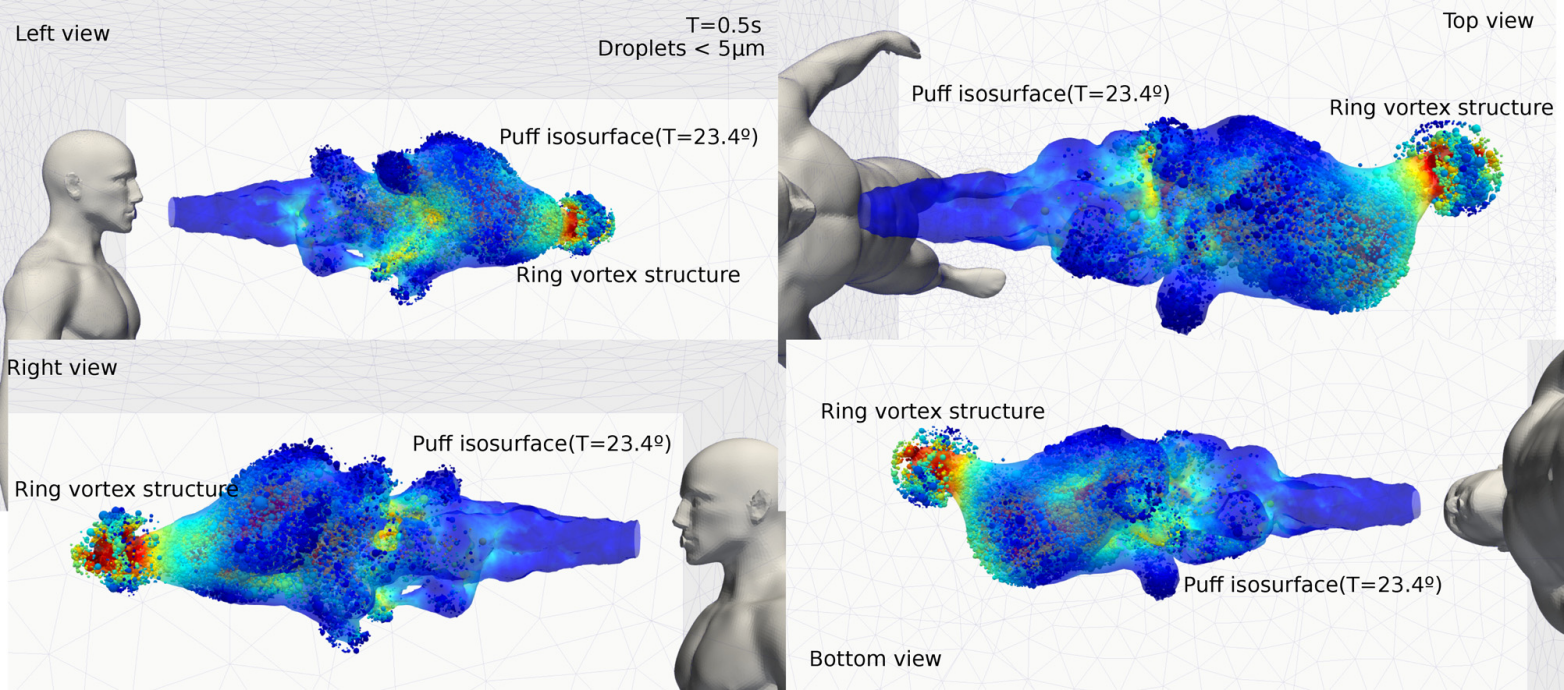
Four different views of the thermal puff. The structure of the warm puff is shown by a temperature iso-surface of 23.4° colored by the velocity of the gas phase. The droplets are represented with the diameter size and colored by the velocity.

Figure 7 shows different views (left, right, top, and bottom) of the thermal puff cloud with droplets under 5 *μ*m diameter. The puff cloud is represented by the isosurface of temperature equal to 23.4 °C colored by the velocity of the gas phase^54^ while the droplets are represented with the diameter size and colored by its velocity. The bulk of the thermal puff remains coherent and exhibits some turbulent eddies, except for the vortex ring structure. Silwal *et al.*^55^ suggested that virus-laden particles tend to remain concentrated within the moving puff cloud after an expiratory event and a direct collision with the traveling plume-front is the likely result in very high virus exposure.

#### 2. Vortex ring structure

**FIG. 8.**
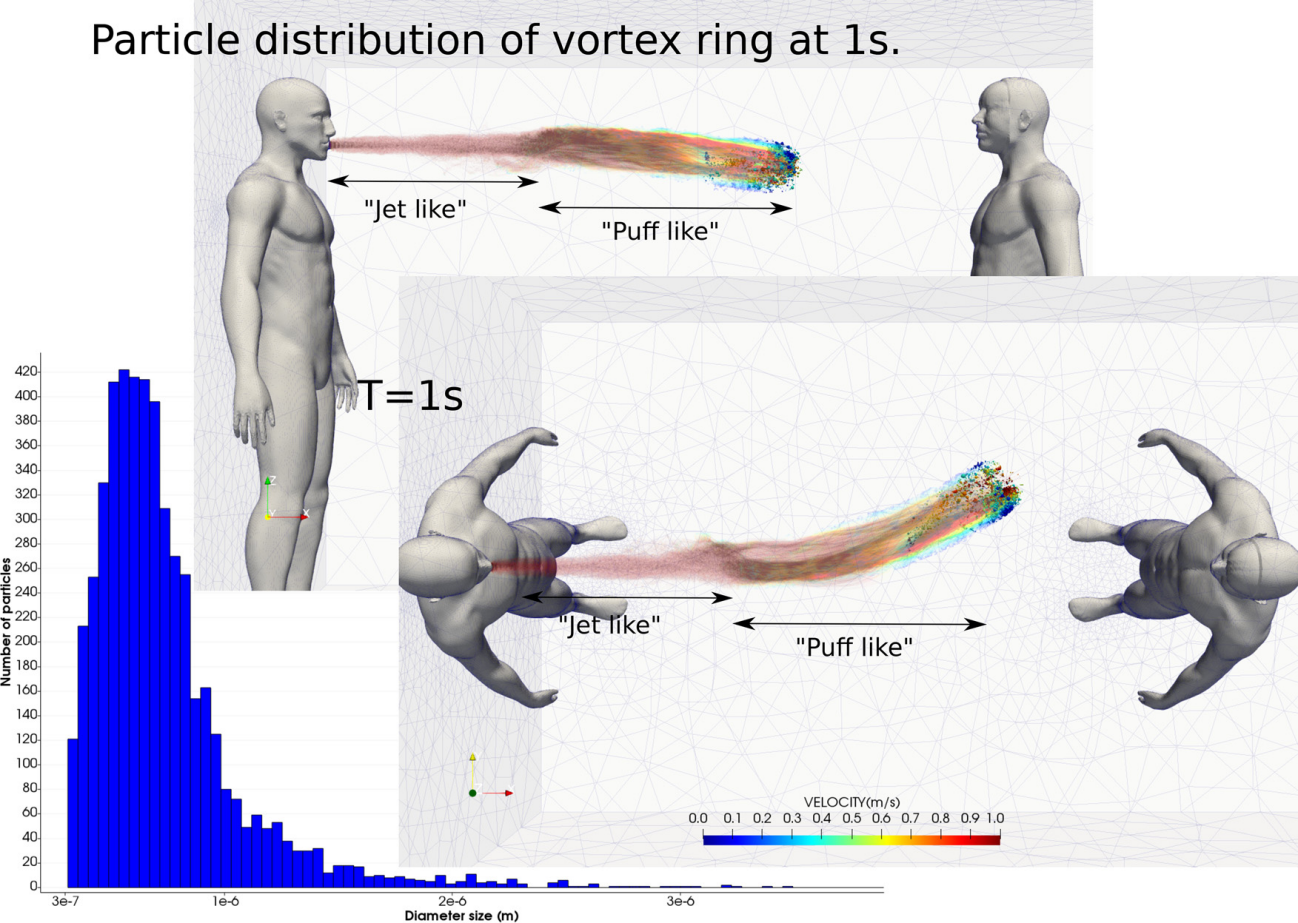
Droplet trajectories of the vortex ring structure at 1 s and the droplet size distribution corresponding.

Vortex structures during the cough are characterized by the well-known vortex ring structures generated at the edge of the jet.^16,53^ We note that expiratory events such as a cough are pulsatile; however, this study considered a cough as a single pulse. Figure 8 shows the droplet trajectories for each droplet found in the vortex ring structure at 1 s. The trajectories represent the individual droplet motion colored by velocity. We observe from the droplet size distribution (Fig. 8) that the majority of the particles are under 1 *μ*m, which represents the tracer-like behavior of low inertia droplets that become entrained by the vortex ring structure and the trailing jet.

Bourouiba *et al.*^21^ demonstrated that the temporal evolution of the vortex ring structure is characterized by two periods. Our results show that the first period (0–0.2 s) is dominated by the cough jet with a high velocity and important fluid-droplet mixture (straight and red pathlines in Fig. 8), while the second period (0.2–1.0 s) is dominated by the cough puff with low velocity represented by the curved mixed colors pathlines in Fig. 8.

The vortex ring structure is deviated to the left side of human-1. This feature was observed in the literature, numerically^54^ and experimentally.^21,56,57^ In this study, we included the nasal anatomy in the second human where the nasal cavity exhibited asymmetry between the left and right cavities, which is well established as the nasal cycling phenomenon. During peak exhalation, two local circular jets exit from the two nostrils, where one will exhibit a faster flow rate due to a more patent airway. In addition, two streams of air are pulled into the nostrils at different flow rates during inhalation. While the cloud puff deviation is relatively random, there is a bias to one side since when the cloud puff reaches the second human, its velocity is very low and is easily influenced by the ambient conditions, including the human thermal plume the respiratory breathing flow.

### B. Post-cough

After characterizing the main features of the cough with a resolved LES simulation using the M1-mesh, the flow field containing the velocity, pressure, temperature, and water vapor fraction was recorded, in addition to the droplet position and its properties. The flow and droplet data fields were interpolated onto the M2-mesh, which was optimized to track the droplet transport between the two manikins without mesh refinement. The mesh provided an increase in computational speed through an increase in the time step of 60 times that of the M1-mesh. We assume no loss of information between the two meshes since the cough at 1 s has transported sufficiently far from the refined cough region. The dispersed cough jet exhibited a low velocity outside the main cough region, where the element sizes were almost identical between the two meshes. After 1 s of simulation, the droplets of interest (<20 *μ*m) reached their droplet nuclei state described by Oliveira *et al.*;^50^ thus, the evaporation mechanism was not taken into account in the post-cough period. Droplets larger than 20 *μ*m are more subjected to gravitational settling. They are less of interest for this study of airborne droplet dispersion over a long period, and in this study, we modeled for 10 min. The simulation over an extended period in tracking the droplet trajectories provides valuable information for prolonged exposure to airborne viruses of a second person at a distance of 2 m away.

**FIG. 9.**
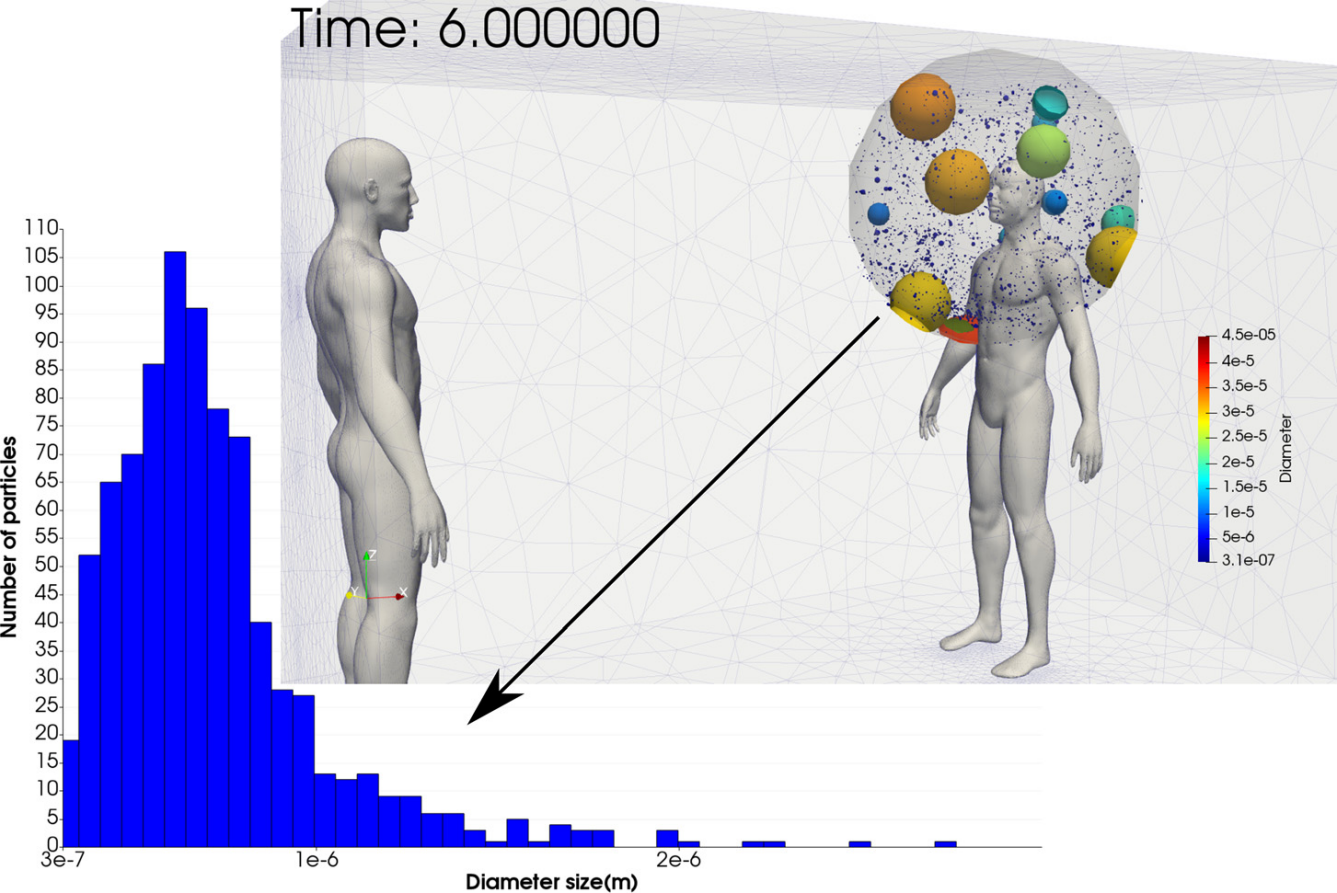
Particle distribution onto the breathing region (0.5 m sphere around the head) of human-2 at 6 min.

**FIG. 10.**
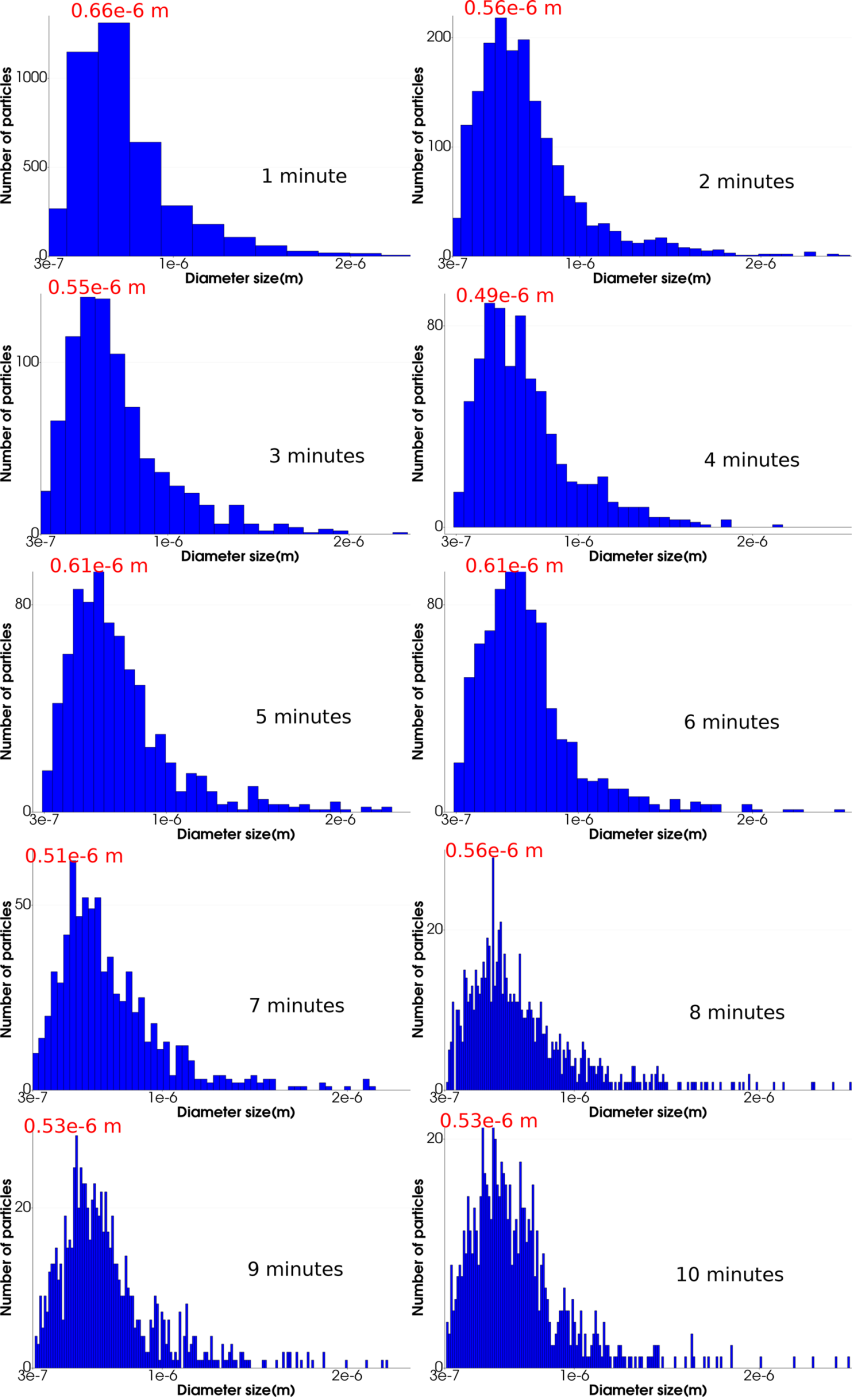
Particle distribution onto the breathing region of human-2 at every minute.

The tidal volume inhaled by the human-2 for each respiratory cycle was 550 ml. Exposure to the cough droplets was determined by defining a breathing region where we set a sphere around the nose with a radius of 0.5 m representing the tidal volume. Figure 9 depicts the droplet distribution found in the breathing region of human-2 at 6 min after the cough. Figure 10 shows the distribution at every minute up to 10 min. The data are useful for understanding the evolution of the droplet size distribution in the breathing region and thus to be able to model the breathing risk exposure. Figure 10 shows the number of droplets that disappear gradually with time, reducing the bin size (number of droplets per bar); thus, the probability distribution analysis is difficult. However, the distribution trend appears constant, which could be described as a unimodal distribution. The red number on the peak at each distribution plot can be considered the unimodal distribution's modal value or mean value. These values confirm that the critical diameter size susceptible to inhalation is 0.5 μm. The breathing cycle of the human-2 was resolved, allowing information of the fate of these droplets located in the breathing region.

**FIG. 11.**
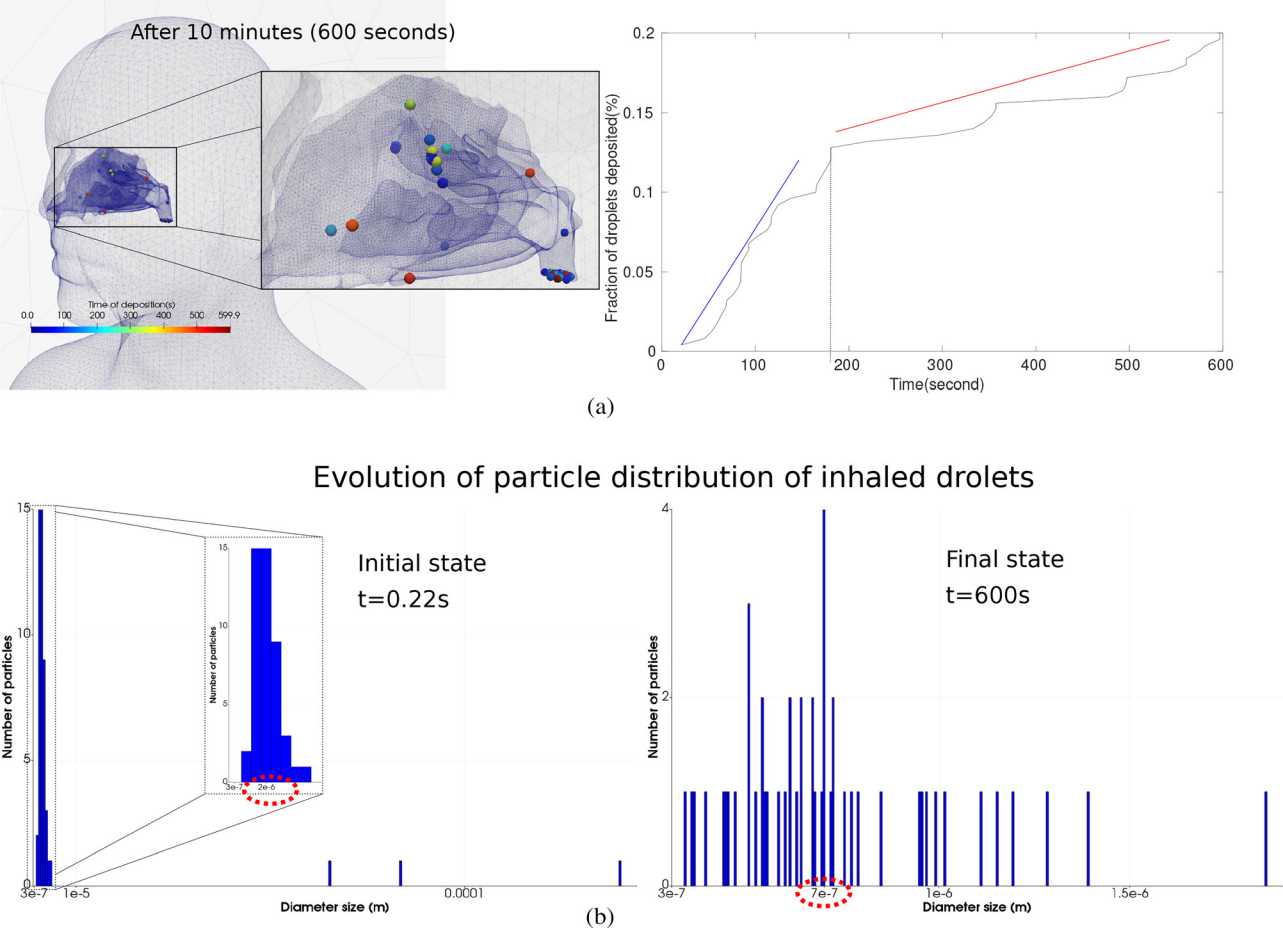
Features of inhaled droplets: (top) droplets inhaled after 10 min with the temporal evolution of the deposition of droplets inhaled; (bottom) initial and final particle distribution of the inhaled droplet after 10 min.

Droplet inhalation into the nasal cavity was monitored, which is referred to as aspiration efficiency or inhalability. The breath volume and rate inhaled influence the flow field in the vicinity of the nostrils. Figure 11(a) shows droplet inhalation and its deposition in the nasal cavity over 10 min and the temporal variation of the droplet deposition for the droplets inhaled. The temporal evolution of the droplet deposition provides insight into the exposure risk shown in Fig. 11(a). Two different gradient lines were observed, the first between 0 and 180 s (depicted by the blue line), which has a sharper gradient due to the influence of the trailing puff from the cough and the vortex ring structure that was deviated. The second gradient between 180 and 600 s (depicted by the red line) exhibited a flatter gradient, correlating with the decaying and dissipated momentum from the cough. The disturbed flow field from the cough returns to its ambient state with natural convection currents and breathing patterns contributing to the flow field.

Each deposited droplet was monitored where the changes in its properties such as the diameter due to the evaporation rate are determined. Figure 11(b) shows inhaled droplet distribution variation at the start of the cough (t = 0.22 s, corresponding to the end of the introduction of the droplets in the computation domain) and at the end of the simulation (at t = 600 s). We observe for both plots a unimodal distribution with a mean value equal to 2 μm just after the cough and 0.8 μm after 10 min. The number of inhaled droplets was low, so the probability distribution analysis was difficult. It is likely due to the limited exposure time of 10 min and the physical distance between the two manikins of 2 m. These observations offer insight into the risk of airborne transmission, demonstrating the importance of the evaporation rate. After 10 min, the inhaled droplets evaporated until 40% of the initial diameter, which affects the aerodynamics of the droplets.

Some limitations of this study include a single specific case of two humans 2 m apart with one ambient condition. A parametric study with combinations of room conditions (temperature and humidity) and number of humans and distances apart was not possible as it is computationally prohibitive and impossible to achieve the results in a realistic time frame to capture the cough in detail and post-cough droplet dispersion in a room for 10 min using large eddy simulation.

A second limitation was our cough analysis that assumed a single pulse; however, a recent study^30^ showed that droplets emitted from later pulses, which originate from deeper in the lung, were transported further due to vortex interactions generated by earlier jet pulses, which may enhance the likelihood of droplets reaching the second human.

## V. CONCLUSION

The uncertainty of the airborne transmission risk of viruses is a crucial health issue. A significant contributor to the spread of the virus is that asymptomatic patients can expel the virus without knowing that they are infected. If we consider the worst-case scenario of the respirable droplet range, then if these droplets become airborne, they are transmissible as they are small enough to be inhaled. Airborne transmission of aerosols requires a multiscale approach that spans different lengths from micrometer-sized droplets to meters in the length of the indoor rooms, while a coughing event is characterized in milliseconds, and convection-diffusion behavior in a room is described over minutes or even hours, if a person is in the same room for a prolonged period. To resolve the multi scales, we applied separate meshes to the computational domain for the separate coughing event for 1.0 s, followed by the airborne transmission of the ejected droplets.

The primary cough characteristic is the persistent puff that is made up of the smallest droplets ( <5 μm). These droplets are first transported by the cough momentum and become airborne as they reduce in diameter while evaporating under the influence of humidity. The evaporation process of these droplets is rapid, completed within 0.5 s, suggesting that a viral-containing droplet will be stripped of its water content, reducing its size to allow it to float through the air, increasing the exposure risk of direct transmission of the virus to another person. Inhalation exposure risk of pollutants is typically evaluated as a longitudinal study, but a short exposure period might be enough for transmission to occur with highly infectious diseases.

Using a separate computational mesh, we modeled aerosol dispersion for 10 min to determine the airborne transmission between two humans standing 2 m apart. Droplet inhalation was also tracked, where the inhalability was dependent on the inhaled volume and flow rate. The amount inhaled was low, presumably due to the sufficient distance between the two humans. We monitored the droplets that came within the breathing region of human-2, which provides an alternative but important metric to exposure risk. The results suggest that within the first 1–2 min, the cough caused the highest exposure and with the exposure decaying with time. The high number of droplets in the breathing region, and yet only a small number of droplets were inhaled, suggests that the microenvironment around the human body has a significant role. As there were no ventilation flows, the natural convection currents and respiratory breathing become important where rising thermal plumes and exhalation breaths remove droplets away from the nostrils. It is noted that the peak in the droplet size distribution within the breathing region showed diameters of approximately <5 μm, which were contained and transported within the cough puff. These submicrometer diameters are a result of evaporation, and the results will differ under different humidity conditions.

This study applied highly resolved modeling of the cough jet dynamics and a prolonged simulation to track aerosol dispersion over 10 min. However, only a single case of two humans with one separated distance was used. Additional scenarios of increased occupancy, ventilation strategies, and environmental conditions can be considered to provide a more holistic approach to optimizing ventilation strategies.

The purpose of this study was to evaluate the critical diameter size susceptible to inhalation between two humans standing 2 m apart in a microenvironment. The multiscale approach of this study offers an accurate airborne transmission model of aerosols taking into account the evaporation aspect. A small number of inhaled droplets by the second human make the probability distribution analysis difficult and limit the study. However, these results help understand the evolution of the droplet size distribution in the breathing region and thus model the breathing risk exposure.

## Figures and Tables

**FIG. 12.**
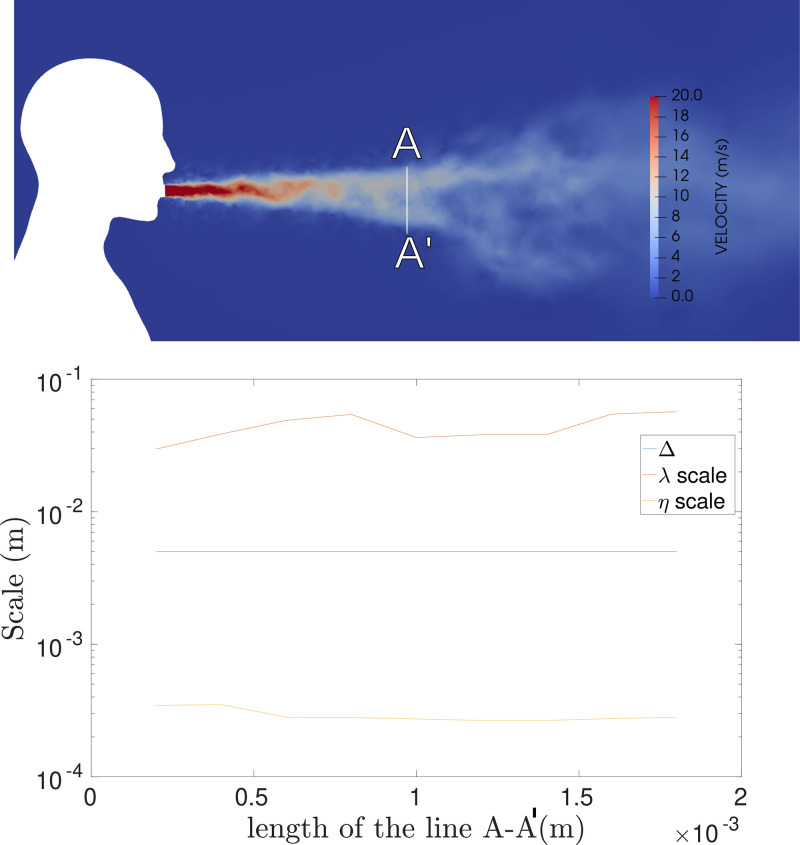
Comparison of different turbulence scales along the A–A′ line at the constant peak value of the cough ( 8 l/s) during t= 0.0 to 0.5 s with mesh M1.

**FIG. 13.**
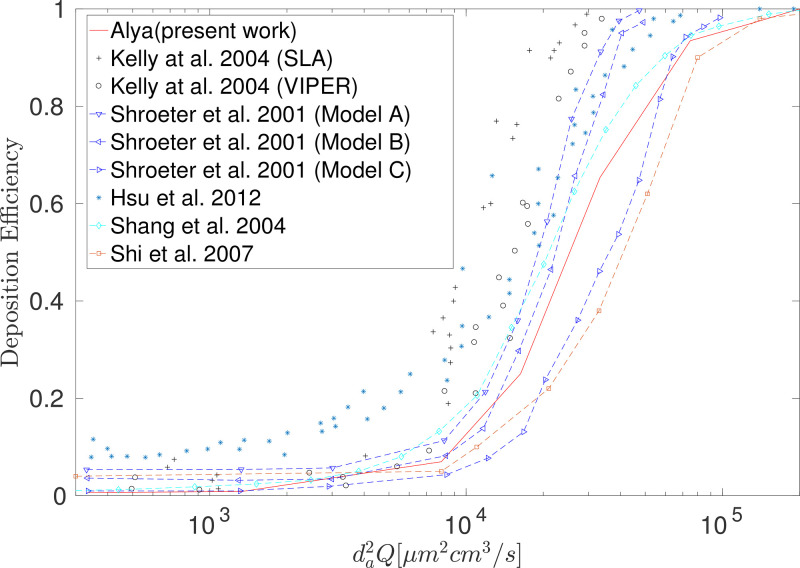
Microparticle deposition efficiency comparison between simulation and experiments.

## Data Availability

The data that support the findings of this study are available from the corresponding author upon reasonable request.

## APPENDIX: VALIDATION

### 1. Grid convergence

In the LES approach, the grid (Δ) and time resolution ( Δt) are critical to ensure a reasonable ratio between the grid size and the smallest scales of turbulent motions (the Kolmogorov scale, *η*). While no universally criterion exists for the LES grid size, a ratio $\frac{\Delta }{\eta }$ less than 20 is reasonable.^35^ The grid resolution should also fall into the turbulent length scales of the Taylor microscale (*λ*) and Kolmogorov scales (*η*). The Taylor microscale is used to characterize a turbulent flow and is larger than the Kolmogorov scale^58^ defined as

$$\lambda =\sqrt{\frac{10(\nu +\nu_{\mathrm{sgs}})\mathrm{TKE}}{\varepsilon }},$$
(A1)

$$\eta ={\left(\frac{{\left(\nu +\nu_{\mathrm{sgs}}\right)}^{3}}{\varepsilon }\right)}^{\frac{1}{4}},$$
(A2)

where TKE is the turbulent kinetic energy, *ε* is the turbulent dissipation rate, *ν* is the fluid kinetic viscosity, and *ν_sgs_* is the subgrid-scale viscosity. Figure 12 compares the grid size (Δ) of mesh M1, with the turbulence scales at the constant peak value of the cough ( 8L/s) during t= 0.0 to 0.5 s. The profiles were taken along line A–A′ which was located in the jet core at 20 D of the mouth opening area. The grid size was between the Taylor and Kolmogorov scale and the equivalent ratio $\frac{\Delta }{\eta }$ was 15, which satisfied the criterion sufficient grid resolution for LES.

In addition to the turbulent length scales, the temporal resolution ( Δt) is also critical to capture the highest frequencies of turbulence. The smallest time scale in a turbulent flow (the Kolmogorov time scale $\tau_{\eta }$) is given as^58^

$$\tau_{\eta }={\left(\frac{\nu +\nu_{\mathrm{sgs}}}{\varepsilon }\right)}^{\frac{1}{2}}.$$
(A3)

Based on the period ( t= 0.0 to 0.5 s) with M1 mesh along the line A–A′, the minimum $\tau_{\eta }$ was equal to $3\times 10^{-4}\;s$. Therefore, the time step used for the LES simulation was $5.0\times 10^{-6}\;s$ (see Table I). The time resolution of the LES simulation was sufficiently small to capture the highest frequencies of turbulence.

### 2. Particle deposition validation

To validate the multi-scale problem deposition, particle deposition in the nasal cavity was used. It was compared to experimental data reported by Refs. 59 and 60 and numerical data^61–63^ (see Fig. 13) where the flow rate used for the comparison was a constant 20 l/min. In order to standardize the results, the inertial parameter (IP) was used

$$IP=d_{a}^{2}\cdot Q,$$
(A4)

where *d_a_* is the particle aerodynamic diameter (i.e., 1 g/cm^3^) and *Q* is the volumetric flow rate. Figure 13 shows good agreement between the simulation performed by the present code (Alya) and the numerical results of Refs. 61–63. Differences in deposition results are due to the coarser airway surfaces in the replica producing higher deposition efficiencies than the numerical model observed in the literature through studies on the “wall roughness enhanced particle capturing effect,”^63,64^ or the effect of surface roughness on deposition.^65^
